# Fabry disease: recent advances in pathology, diagnosis, treatment and monitoring

**DOI:** 10.1186/1750-1172-4-21

**Published:** 2009-10-11

**Authors:** Björn Hoffmann

**Affiliations:** 1 Dept for General Pediatrics, University Children's Hospital, Heinrich-Heine-University Düsseldorf, Germany

## Abstract

**Background:**

In Fabry disease (α-galactosidase A deficiency) accumulation of Globotriaosylceramide (Gb3) leads to progressive organ failure and premature death. The introduction of enzyme replacement therapy (ERT) was the beginning of a new era in this disorder, and has prompted a broad range of research activities. This review aims to summarize recent developments and progress with high impact for Fabry disease.

**Methods:**

A Pubmed analysis was performed using the search terms "Fabry disease", "Anderson-Fabry disease", "alpha-galactosidase A" and "Gb3". Of the given publications by 31st January 2009 only original articles recently published in peer reviewed journals were included for this review. Case reports were included only when they comprised a new aspect. In addition we included relevant conference abstracts when the results had not already been published as original articles.

**Results:**

Apart from Gb3-accumulation cellular and organ specific damages may be related also to inflammatory and immunological consequences. It will be interesting whether this may lead to new therapeutic strategies in the treatment of Fabry disease. Since newborn screening is still difficult in Fabry disease, detection of patients in populations at risk is of great importance. Undiagnosed patients with Fabry disease may still be found in cohorts of subjects with renal diseases, cardiomyopathy and TIA or stroke. Efforts should be undertaken to identify these individuals and initialise ERT in order to hault disease progression. It has also been demonstrated that Gb3-accumulation leads to pre-clinical damages and it is believed that early treatment may be the only possibility so far to prevent irreversible organ damage.

## Introduction

The introduction of enzyme replacement therapy (ERT) for Fabry disease in 2001 was the beginning of a new era for this lysosomal storage disorder. More than 2000 patients have been treated with either one of the two available formulations of genetically engineered α-galactosidase A. In general, safety and efficacy of both, Agalsidase alfa (Replagal, Shire Human Genetic Therapies) and Agalsidase beta (Fabrazyme, Genzyme Corp.), have been demonstrated [1,2]. The introduction of these drugs was not only the desired hope for patients. It broke also new ground in research including a better understanding of pathology, natural course of the disease, treatment and outcome. Before the introduction of ERT for Fabry disease it had already been known that the basis of this disorder is the X-linked deficiency of the lysosomal enzyme α-galactosidase A [3]. The accumulation of globotriaosylceramide (Gb3) had been regarded as the key link between pathology and clinical symptoms. Typical manifestations had been described as angiokeratoma, sweating disturbances, neuropathic pain, and hearing impairment. In addition, affection of the heart, the kidney and the central nervous system had been seen [4,5].

To keep up with newest research in such a developing field is important, but may also be difficult. This review aims to summarize recent developments in the understanding of pathology, symptomatology, diagnosis, and treatment effects in Fabry disease. A particular focus is brought on publications appeared in 2008 as some of them are of outstanding importance and high impact.

## Methods

A Pubmed-research was undertaken by the end of January 2009 for publications covering all aspects of Fabry disease. Search terms were 'Fabry disease', 'Anderson-Fabry-disease', 'alpha-galactosidase A', 'enzyme replacement therapy', 'globotriaosylceramide', 'Gb3' and 'lysosomal storage disease'. For this review, only recently published original articles have been considered. Case reports were included only if they described new findings. In addition to this, we included available abstracts from international conferences if the corresponding publications have not yet been appeared in peer reviewed journals.

## Results

Overall, 37 publications from different peer reviewed journals and 9 conference abstracts were appraised for this review. We classified these publications into eight categories focussing on (1) pathology, (2) heart, (3) neurology, (4) kidney, (5) children with Fabry disease, (6) diagnosis, (7) treatment, and (8) monitoring in Fabry disease.

### Pathology

The absence of infantile manifestations of Fabry disease despite deficient α-galactosidase A and Gb3-accumulation supports the hypothesis that Gb3 is not solely responsible for disease manifestation. However, Valbuena et al. hypothesized that the overloading of lysosomes with Gb3 may simply lead to the rupture of cytoplasma and in consequence to cell death [6].

Secondary effects of Gb3 accumulation which might be responsible for disease pathology include also inflammatory processes [7]. Recently, Gb3 has been shown to be identical with membranous CD77 [8] which is supposed to play an important role in apoptosis and necrosis [9]. In addition, Rozenfeld and co-workers reported perturbed leukocyte function in Fabry disease compared to healthy controls and abnormal numbers of different immune cells, including lymphocytes, monocytes, CD8+ cells, B cells and dendritic cells [10]. Noteworthy, in another study no correlation was found between CRP-levels as a global inflammatory marker and the Mainz-Severity-Score-Index (MSSI) as a marker for disease severity [11].

Other authors reported on auto-immunological reactions in α-galactosidase A deficiency. Moore et al. found higher expressions of neuronal apoptosis inhibiting protein as a key anti-apoptotic mediator in children with Fabry disease compared to healthy controls [12]. Noteworthy, this upregulation did not change after institution of ERT, whereas apoptosis inducing factor appears to be upregulated under ERT. The authors were not able to explain their observations, but further research has to be directed on this topic.

Finally, Gb3-accumulation has been reported to induce oxidative stress and/or the formation reactive oxygen species (ROS) [13]. Lipids and proteins may be oxidised and may be unable to function. Of note, ERT increased the generation of ROS *in vitro*, and up-regulated the intracellular adhesion molecule ICAM 1. The authors hypothesized that ERT may enhance endothelial damage, allowing to understand continuously occurring strokes in patients on treatment.

Another gateway into alteration of endothelial function may be given by the Nitric-Oxide-Synthase-3-genotypes. Endothelium-derived nitric oxide is a key regulator of vessel wall function and cardiovascular homeostasis. A genetic variant of the Nitric-Oxide-Synthase-3-gene is suspected to disturb this homeostasis and has been found to be associated with a decreased thickness of the posterior wall of the left ventricle [14]. This observation may in part explain the large variability of cardiac phenotypes in Fabry disease.

A so far unknown observation of Gb3-accumulation has been made by Wang and colleagues who described Gb3 storage in pulmonary smooth muscle cells and vascular endothelium of a female patient with Fabry disease [15]. This finding offers an explanation for dyspnoea in the context of Fabry disease apart from cardiac involvement. Four years of ERT in the 51-years old woman led to significant clinical improvement of pulmonary symptoms and in part improved lung function. However, CT-scans continuously showed fibrotic alterations.

#### Heart

Despite the high frequency of cardiomyopathy in Fabry disease [16], only little is known on the mechanisms leading to functional impairment and on the early cardiac affection.

To identify early cardiac abnormalities Toro and colleagues performed Tissue Doppler Imaging (TDI)-studies in 59 adult patients and compared these results with that of 24 healthy controls [17]. The authors demonstrated significant abnormalities of systolic and diastolic ventricular function with TDI even in patients with normal conventional echocardiography. In addition, isovolumetric contraction time was found to be the most sensitive parameter for detecting these functional changes.

Consistent with this, Kampmann et al. reported on cardiac manifestations of Fabry disease in 20 children below the age of 18 years prior to ERT [18]. Thirty-five percent of the children already had left ventricular hypertrophy, and the remainder subjects had a LVM-index above the 75th percentile of healthy controls. During the 26-months follow-up period the LVM-index increased in more than 85% of the patients, while the remainder patients entered the echocardiographic criteria for LVH. Noteworthy, the increase in LVM was not followed by abnormalities in either systolic or diastolic function, and in general there were no signs of LVH seen in ECG. Another warranting observation of this study was the demonstration of reduced heart rate variability (HRV) in boys with Fabry disease suggestive of an impaired autonomic control of the heart which may be responsible for the increased cardiac morbidity. Palecek et al. demonstrated that also affection of the right ventricle is a common finding in both, males and females with Fabry disease [19]. Impaired diastolic function was observed in about 50% of the patients, and the authors speculated that involvement of both ventricles may reflect the progressive character of the disease.

Weidemann et al. stressed the involvement of the heart valves in particular in patients with advanced cardiomyopathy, where insufficiencies of the mitral, aortic and tricuspid valve were found [20]. It is likely that the altered myocardial morphology in advanced cardiomyopathy maybe together with fibrosis of cardiomyocytes may indirectly lead to heart valve regurgitation.

Apart from fibrosis of the myocytes, disturbances of the myofilamental structure has been observed in isolated cardiomyocytes of male patients with Fabry disease and LVH [21]. A high percentage of Gb3 was found widely distributed over the cells, and it is not unlikely that the storage material directly impairs ventricle function (e.g. by impaired relaxation or stiffening of cardiomyocytes). The authors hypothesised that such changes may be responsible for diastolic dysfunction in Fabry disease. On a more functional level, Chimenti and co-workers found that the maximal isometric tension of a single cardiomyocyte was reduced [21]. This reduction correlated with the decreased systolic velocities from TDI and with the degree of myofibrillolysis. In addition, myofibrillolysis was associated with degradation of myofilamental proteins suggesting that cardiomyocyte impairment may in part be explained by proteolysis. Progression of Fabry disease cardiomyopathy may be related to fibrosis of endomyocardial tissue, but was not correlated with TDI measurements of systolic or diastolic LV function in this study.

Cardiac involvement in Fabry disease is one of the major causes for premature death in Fabry disease [4]. However, it is generally accepted that classic myocardial infarction as a result of atherosclerotic coronary heart disease is rare. Coronary angiographic properties were determined by intravascular ultrasound in nine patients and ten atherosclerotic controls [22]. Atherosclerotic plaques in Fabry disease were suspected to have a larger number of lipid cores, and the pattern of the lesions was more echogenic. The authors speculated that Gb3 and disease-specific trophic alterations may explain the differences to regular atherosclerotic formations.

In a double-blind, randomized, placebo-controlled study [23] sixteen adults were followed over a period of six months before 10 of them entered a two years extension study. The authors found a mean decrease in myocardial Gb3-content of ~20% in the treated group whereas the placebo-group showed a mean increase in Gb3 of ~10% [23]. Though reduction of myocardial Gb3-content did not reach significance, LVM as measured by cardiac MRI and echocardiography was significantly decreased in the patients treated with Agalsidase alfa compared to an increase in the placebo group. After two years of treatment these results were confirmed by repeated cardiac MRI. Ejection fraction as a measure of cardiac function remained normal or hypernormal in the entire cohort. Shortening of QRS-duration confirmed previous reports on normalized conduction [2]. The authors indicated that the significant reduction in LVM may be clinically relevant since LVM has been demonstrated to be an independent risk factor for premature mortality in other conditions.

From an observational study it has been reported that ERT with Agalsidase beta may reduce LVM and interventricular septum thickness [24]. In contrast to this Koskenvuo et al. reported only minimal effects of Agalsidase beta on cardiac symptoms of nine patients with Fabry disease who were enrolled in an open-lable study [25]. Only resting heart rate was significantly decreased over a follow-up period of 2 years, but no improvement in echocardiographic measures, ECG or exercise capacity was observed. Improvements seen in stroke volume on cardiac MRI after 12 months ERT were not retained after two years, and no other parameter reached significance.

Echocardiographic follow-up over more than three years in 29 males and females with Fabry disease under ERT with either of the available Agalsidase formulations failed to prove a decrease in left ventricular wall thickness, or left atrial size though improvement of diastolic function was observed [26]. However, it has to be noted that the age of the study population was relatively young (37 ± 12 years) and that no further progression of the disease was observed in respect to cardiac manifestations under ERT. This observation is supported by a study from Kampmann et al. on the natural course of cardiomyopathy in male and female patients with Fabry disease [27]. Repeated echocardiography was performed over an average time of 4.5 years in 76 patients. There, left ventricular mass index increased with age in both, males and females, respectively. Median time of onset of left ventricular hypertrophy was 44 years in males and 55 years in females. As expected, LVH was more frequently found in male patients and was also related to age. No functional measure was related to left ventricular mass index or left ventricular hypertrophy. Of note, left ventricular mass index increased by some 4 g/m^2.7^*year in men and slightly more than 2 g/m^2.7^*year in women. Following the authors, this difference may be explained by the mosaic-like distribution of Gb3 in female cardiomyocytes compared to male patients. In summary, these studies recommend initiation of ERT at an early stage of the disease since at least some of the cardiac manifestations may be irreversible [26,27].

Recent data showed that left ventricular wall thickness showed significant improvement over a period of eight years in patients under ERT with Agalsidase alfa [28].

Apart from morphologic improvement of LVH and cardiac mass, Lobo and co-workers tried to evaluate a clinical effect of ERT on cardiac function by performing a 6-minute-walk-test and bicycle stress testing [29]. However, the authors mixed male and female patients despite the different clinical affection. Moreover, females were on ERT only one third of the time males were treated with one of the enzyme formulations. In addition, both tests used may be significantly biased, e.g. by motivation, neuropathic pain in feet or aggravation of pain under physical exercise.

#### Neurology

Probably the most debilitating symptom in Fabry disease is neuropathic pain. Though often labelled as "acroparaesthesia", it is well known today that virtually the whole body can become place of painful experiences [30].

The typical neurophysiologic pattern of pain in Fabry disease allows discrimination from other sensory neuropathies. Standardized quantitative sensory testing demonstrated severe impairment of thermal discrimination compared to patients with sensory neuropathies of large nerve fibres and to patients with other small fibre neuropathies [31].

Apart from the peripheral nervous system, the central nervous system is also known to be affected in Fabry disease, too. Cerebrovascular events significantly contribute to the pre-term mortality of Fabry disease with a median cumulative survival time of 50 years [4]. Based on clinical-neurological and MRI evaluation Buechner et al. reported on a prevalence for CNS-involvement in up to 72% of patients with Fabry disease [32]. Cerebrovascular disease occurred at a mean age of 42 ± 11 years in males and approximately 10 years later in females. In contrast to other reports, the authors found a nearly equal distribution of cerebrovascular involvement between vertebrobasilar and carotid districts.

Alari et al. observed changes in cerebral blood flow velocities (CBFV) in patients with Fabry disease who were treated with ERT [33]. At baseline, there was a tendency towards higher CBFV in patients with Fabry disease compared to age-matched healthy controls. After 30 months ERT CBFV decreased significantly, which is in good agreement with previous observations over a shorter period of time [34].

Neuropsychiatric symptoms in Fabry disease were evaluated by Schermuly et al. together with MRI and diffusion tensor imaging [35]. Patients with Fabry disease were significantly more depressed and showed a lower cognitive performance than healthy controls. Interestingly, pain interference was significantly correlated to white matter lesions supporting previous observations by Cole et al. [36].

#### Kidney

Close examination and laboratory analyzes is important to detect also pre-clinical affection of the kidney in Fabry disease. Tøndel et al. [37] performed kidney biopsies in nine children, including two boys under ERT. Accumulation of Gb3 was found irregardless of clinical nephropathy, and structural changes of the kidney were observed in nearly 80% of the patients including glomerulosclerosis and interstitial fibrosis already in these young patients. Moreover, changes were seen in the glomerulum, the tubulo-interstitial space and small renal vessels. Noteworthy, the two boys receiving ERT developed de novo albuminuria despite treatment. The authors suggested kidney biopsies in all children and adolescents with Fabry disease and albuminuria in order to evaluate the extent of renal damage.

Erroneously, for decades females with Fabry disease were considered as carriers only. Meanwhile, there is no doubt that they are in fact patients who may develop the whole spectrum of the disease [5,38].

A cross-sectional analyzes of the Fabry Registry focussing on kidney involvement in females revealed that renal impairment occurs in all classes of the chronic kidney disease classification including end-stage renal disease [39]. Consistent with this other authors found proteinuria in one third of the female and nearly two third of the male patients, increasing with age in both genders. The latter finding is in contrast to a previous report by Deegan et al. [40], but was confirmed by other authors [6]. Kidney affection was clearly evident by Gb3-accumulation in every glomerular cell type, and accumulation in peritubular capillaries was grossly related to the severity of nephropathy. These changes were observed in an early stage of the disease, and the authors speculated on its value as an indicator for ERT efficacy. In addition, non-specific glomerular and tubulo-interstitial lesions of degenerative origin were also seen. The authors concluded that kidney involvement in females with Fabry disease is not different from findings in male patients.

Rather seeking for morphologic but functional alterations in patients with Fabry disease, Vylet'al and co-workers reported on quantitative and qualitative changes in the excretion of uromodelin (UMOD) [41]. Attenuated UMOD-expression was suspected to be directly linked to lysosomal storage processes as both, UMOD and accumulated Gb3 meet in the Henle-Loop of the kidney. Deficient UMOD-expression has been reported in association with impaired tubular function, in particular with impaired urine-concentration. Hence, the reported observations may explain the changes in tubular function seen in Fabry disease, and in particular the phenomenon of hyperfiltration reported in some patients.

Treatment effects have been analyzed based on data from 165 patients with Fabry disease enrolled in the Fabry Outcome Survey [42]. Feriozzi et al. demonstrated that ERT with Agalsidase alfa may slow the decline in renal function of male and female patients. Moreover, this effect was also seen in patients with advanced renal failure (eGFR ≤ 59 ml/min*1.73 m^2^), and it was sustained over a period of three years.

Both, diagnosis and monitoring of kidney involvement in Fabry disease is hindered by the repeated discrepancies between estimated glomerular filtration rate (eGFR), 24-hour urine creatinin clearance (Cr) and isotopic GFR measurement.

#### Paediatric Fabry disease

An observational study on children enrolled in the Fabry Registry characterized disease manifestation in 352 children (194 boys, 158 girls) before the institution of ERT [43]. In this cohort reported age at onset of symptoms was 6 years in boys and 9 years in girls, respectively. The most frequent symptom was neuropathic pain which was reported in about 60% of the boys and some 40% of the girls. Gastrointestinal symptoms were frequently reported with more than 1/4 of patients affected also on follow-up (baseline definition not clear). The frequency of abnormal skin findings (i.e. Angiokeratoma) in this group of patients was about 25% for abnormal sweating, and slightly more for abnormal heat tolerance. Abnormal cold intolerance was documented in 10-18% of patients (females vs. males). Cardiac involvement included mainly cardiac valve dysfunction (~19% of patients) and arrhythmias (~5% of patients). In addition, conduction disturbances were also seen. Renal insufficiency was reported in 3/144 patients with information on eGFR. The increased frequency of hyperfiltration may be due to an overestimation of eGFR in patients with chronic kidney disease. However, it is also not unlikely that hyperfiltration is an early sign of kidney affection in the context of Fabry disease. Of note, the information regarding quality of life and pain assessment in this study have to be taken with caution since both questionnaires (SF-36 and Brief Pain Inventory) are validated for adults, but not for children.

### Diagnosis

Several studies on the natural history of Fabry disease as well as analyses of the two available patient registries have shown that diagnosis often is carried out more than a decade after onset of first symptoms [44,45]. Indeed, there are patients with a diagnosis not before irreversible organ damage has taken place. Several authors suggested early institution of ERT in order to prevent irreversible organ damage.

Projects are ongoing to evaluate the feasibility of newborn screening for Fabry disease, and pilot studies have been taken place in several countries [46,47]. Males may be easily detected by determination of α-galactosidase A from dried blood spots, but there are still difficulties in detecting females with this method. Determination of residual enzyme activity in females may deliver erroneously normal activities in up to 40% of females with Fabry disease despite significant clinical disease [48].

However, newborn screening for Fabry disease may once be incorporated into already existing screening programs using tandem-mass-spectrometry. Such an implementation would not only allow early diagnosis of Fabry disease, but may also provide valid information on the real prevalence of Fabry disease.

This may be even more important since the pilot studies in newborn screening as well as secondary screening of populations at risk suggested a much higher prevalence than previously reported [1,49]. In Austria screening of male kidney transplant recipients revealed an incidence of 1:262 in this population [50], which is markedly higher than previous studies on a similar cohort [51,52], but less than reported by Nakao et al. several years earlier in patients undergoing haemodialysis [53]. Porsch et al. detected two patients with deficient α-galactosidase A activity out of 577 male patients with unknown reason for renal failure and haemodialysis [54]. The authors agreed that Fabry disease is still under-diagnosed also among patients on dialysis, and they emphasized the importance of proper medical work-up in patients with end-stage renal disease or renal failure.

### General considerations regarding ERT

Providing the missing enzyme to patients with Fabry disease sounds easy and in fact is simple to perform. Confirming previous reports, Keslová-Veselíková et al. reported that intravenously administered enzyme reaches the lysosome via Mannose-6-phosphate-receptor mediated uptake [55]. The uptake of Agalsidase beta into the cells was higher than in Agalsidase alfa, a fact attributed to the enrichment with mannose moieties in Agalsidase beta. In both enzymes a total clearance of the fibroblastic lysosomes from Gb3 was observed after 24 h, and total quantitative analyzes of this clearance revealed a dose-dependency. However, the consumption of Agalsidase beta within the fibroblasts was much faster compared to Agalsidase alfa. The different pattern of enzyme consumption in different cell types and tissues support the idea of early treatment in Fabry disease in order to prevent functional organ damage. Noteworthy, uptake of Agalsidase into endothelial cells was better compared to uptake e.g. into cardiocytes and perineural cells. Hence, for some tissues it may be more effective to prevent Gb3 accumulation rather than cleave the storage material after years. Such a preventive effect may be needed in particular in cells after mitosis and cells with low potential for regeneration, e.g. myocardial cells. However, clinical studies on these effects are lacking.

Though several authors suggested early treatment of patients with Fabry disease using ERT, no randomized double-blind placebo-controlled studies have been performed in children, and most information in the paediatric age group results from observations based on the two clinical surveillance programmes. Anyhow, Ries et al. have evaluated the pharmacokinetic profile of Agalsidase alfa and have shown that this is comparable with that in adult patients [56]. Moreover, Wraith et al. evaluated safety and efficacy of Agalsidase beta in children aging 8-15 years in an open-label study [57]. There, ERT resulted in a decrease of Gb3-accumulation in skin biopsies and improvement of gastrointestinal symptoms after 24 weeks treatment. Interestingly, the authors did not find renal or cardiac symptoms at baseline, and stabilisation was reported under ERT. Forty percent of the patients experienced infusion associated reactions, and nearly 70% of patients developed IgG-antibodies.

Vedder et al. found that α-galactosidase-A-antibodies in adults occurred in about every third patient, but twice as often in Agalsidase beta compared to Agalsidase alfa [58]. Confirming previous observations and in agreement with current understanding of pathology in females, no female patient developed antibodies. Of the patients with neutralizing antibodies more than 75% were treated with Agalsidase beta, and the authors found a correlation between antibody formation and dosage, but no correlation between antibodies and residual enzyme activity/GLA-mutation. In contrast to previous reports [2,59] and independent from the administered enzyme, no decline of antibody levels was observed after 24 months. Clinical parameters such as renal function did not change over a follow-up period of 12 months in any of the treatment groups, and were not related to antibody formation. A decrease in LVM was reported only for 1.0 mg/kg Agalsidase beta, however, the combination of patients who received 0.2 mg/kg Agalsidase beta with those receiving 0.2 mg/kg Agalsidase alfa may be unfortunate as both drugs are different from each other [60]. The authors concluded that high dose treatment may be more effective in reducing Gb3-levels, however, following treatment failure seven patients in this cohort received increased dosages of Agalsidase during the study, and five of them developed worsening of ischemic lesions in the CNS. Hence, a higher dosage may overcome antibody formation, but may not necessarily lead to clinical improvement. In addition, the authors stated that a translation to clinical efficacy is difficult to make as there are no reports available on the clinical outcome of patients with Fabry disease receiving enzyme replacement therapy in relation to antibody formation.

Apart from ERT there is another treatment strategy currently under investigation that is chaperone therapy. Chaperones are able to increase the residual activity in affected individuals, and as they are small molecules they are supposed to cross the blood brain barrier. Shin et al. reported on an association between genotype and response to chaperone therapy [61], a fact that may not surprise. The effect of chaperone therapy is, e.g. to correct the structure of misfolded proteins which in their faulty configuration may not be effective. Hence, only those patients with misfolded enzyme products as a consequence of *GLA-*mutations may benefit from chaperone treatment.

### Monitoring

Both, the pattern of clinical symptoms as well as the response to ERT is heterogeneous and not foreseeable. A biomarker that is uniformly found throughout male and female patients representing the total load of Gb3-storage throughout the body would be very helpful. Ideally, such a biomarker should allow also diagnosing patients independent of their gender.

In this context, Aerts and co-workers reported on the occurrence of lyso-Gb3 in plasma of patients with Fabry disease [62]. Lyso-Gb3 is structurally similar to Gb3 which could not be identified in plasma from control individuals. In hemizygotes a strong correlation between plasma Gb3 and lyso-Gb3 was found, but there was no correlation observed between lyso-Gb3 and the Mainz-Severity-Score-Index as a clinical index for disease severity in male patients. The latter finding may not necessarily limit the impact of lyso-Gb3 but may be related to methodological concerns regarding the MSSI as a cross-sectionally used severity score. However, the MSSI has recently been shown to correlate in a more longitudinal setting with changes under ERT [63]. Lyso-Gb3 in females correlated with the MSSI and with left ventricular mass. In a cell culture, lyso-Gb3 promoted proliferation of smooth muscle cells when given in concentrations similar to those found in plasma of symptomatic patients with Fabry disease. In contrast to Gb3, lyso-Gb3 influenced vascular remodelling *in vivo*, and ERT with either form of Agalsidase lead to decreased concentrations of lyso-Gb3 over a period of one year, but not to complete absence of the compound. In summary, lyso-Gb3 cannot be used as a biomarker.

Another approach for a biomarker was presented by Thomaidis and co-workers, who suggested membranous CD77, the membranous form of Gb3 [8]. The amount of membranous CD77 shall mirror the tissue load of Gb3, and it has been shown to decrease under ERT with Agalsidase alfa in a kidney cell model. Since CD77 may play an important role in apoptosis and necrosis [9], there may be a link to kidney failure in patients with Fabry disease and accumulation of Gb3.

## Conclusion

Important progress has been made regarding the understanding of disease pathology, diagnosis and treatment effects in Fabry disease. Obviously, Gb3-accumulation alone is not responsible for the cellular and organ specific consequences. In fact, inflammation and immunological aspects have to be considered in the development of Fabry disease, too. It remains open whether or not these findings may lead to future treatment options.

Selective screening of populations at risk have clearly shown that there are still undiagnosed patients with Fabry disease, and efforts should be undertaken to identify these individuals, e.g. in cohorts of patients with cardiomyopathy, unexplained renal disease and/or populations with stroke. This is even more important since there is good evidence that ERT may at least halt progression of Fabry disease. Moreover, the publications reviewed here demonstrate pre-clinical damages of heart, kidney and CNS. If there is, however, the possibility that ERT in Fabry disease may prevent irreversible organ damage as it is suggested by some authors and believed by experts in this field, treatment has to be started in young age.

## Conflict of interests

Dr. Hoffmann received travel grants and honoraria for lectures from Genzyme Corp. Germany, from Shire Human Genetic Therapies Germany and Biomarin Germany. There are no conflicts of interest to be declared.
